# EnROL: A multicentre randomised trial of conventional versus laparoscopic surgery for colorectal cancer within an enhanced recovery programme

**DOI:** 10.1186/1471-2407-12-181

**Published:** 2012-05-16

**Authors:** Robin H Kennedy, Anne Francis, Susan Dutton, Sharon Love, Sarah Pearson, Jane M Blazeby, Philip Quirke, Peter J Franks, David J Kerr

**Affiliations:** 1 St Mark’s Hospital, Harrow, UK; 2 Oncology Clinical Trials Office, Dept of Oncology, University of Oxford, Oxford, UK; 3 Centre for Statistics in Medicine, Dept of Oncology, University of Oxford, Oxford, UK; 4 Academic Unit of Surgical Research, School of Social and Community Medicine, University of Bristol and University Hospitals Bristol NHS Foundation Trust, Bristol, UK; 5 Leeds Institute of Molecular Medicine, University of Leeds, Leeds, UK; 6 Centre for Research & Implementation of Clinical Practice, London, UK; 7 Nuffield Dept of Clinical Laboratory Sciences, University of Oxford, Oxford, UK

**Keywords:** Laparoscopy, Colon cancer, Rectal cancer, Enhanced recovery programme, Fast track surgery, Health economics, Cosmetic assessment, Fatigue, Randomised controlled trial, EnROL

## Abstract

**Background:**

During the last two decades the use of laparoscopic resection and a multimodal approach known as an enhanced recovery programme, have been major changes in colorectal perioperative care. Clinical outcome improves using laparoscopic surgery to resect colorectal cancer but until recently no multicentre trial evidence had been reported regarding whether the benefits of laparoscopy still exist when open surgery is optimized within an enhanced recovery programme. The EnROL trial (Enhanced Recovery Open versus Laparoscopic) examines the hypothesis that laparoscopic surgery within an enhanced recovery programme will provide superior postoperative outcomes when compared to conventional open resection of colorectal cancer within the same programme.

**Methods/design:**

EnROL is a phase III, multicentre, randomised trial of laparoscopic versus open resection of colon and rectal cancer with blinding of patients and outcome observers to the treatment allocation for the first 7 days post-operatively, or until discharge if earlier. 202 patients will be recruited at approximately 12 UK hospitals and randomised using minimization at a central computer system in a 1:1 ratio. Recruiting surgeons will previously have performed >100 laparoscopic colorectal resections and >50 open total mesorectal excisions to minimize conversion. Eligible patients are those suitable for elective resection using either technique. Excluded patients include: those with acute intestinal obstruction and patients in whom conversion from laparoscopic to open procedure is likely. The primary outcome is physical fatigue as measured by the physical fatigue domain of the multidimensional fatigue inventory 20 (MFI-20) with secondary outcomes including postoperative hospital stay; complications; reoperation and readmission; quality of life indicators; cosmetic assessments; standardized performance indicators; health economic analysis; the other four domains of the MFI-20. Pathological assessment of surgical quality will also be undertaken and compliance with the enhanced recovery programme will be recorded for all patients.

**Discussion:**

Should this trial demonstrate that laparoscopic surgery confers a significant clinical and/or health economic benefit this will further support the transition to this type of surgery, with implications for the training of surgeons and resource allocation.

**Trial registration:**

ISRCTN48516968.

## Background

During the last 20 years laparoscopic colorectal cancer resection has developed, improving short term clinical outcomes [1-3] while providing equivalent oncological results [4-8]. Some authors [9-11] have reported 3–4 day median postoperative hospital stays after laparoscopic surgery, whereas previously it would have been >10 days [12,13] . Systematic reviews [1,2] document improvements in short term outcomes after laparoscopy, particularly reduction in complications, postoperative pain and hospital stay. However despite these reports the proportion of elective colorectal resections performed laparoscopically in England during 2011 was still only estimated to be 33% (personal communication, MG Coleman)

The last 12 years have also seen the appearance of Enhanced Recovery care or Fast Track Surgery. This applies a multimodal approach to improving care using evidence based interventions and has even resulted in mean and median postoperative hospital stays of 3 days [9,14,15]. Enhanced Recovery care involves preconditioning of patient expectation, modification of surgical technique with smaller incisions, improved anaesthetic and fluid replacement practices, better postoperative pain control, omission of opiates where possible, avoidance of drains and nasogastric tubes, early removal of urinary catheters, mobilization and feeding immediately after surgery, early discharge, and expedited review when necessary. As a result of these developments trials on enhanced recovery care report reductions in postoperative complications and hospital stay [16-18]. There has understandably been great interest in the introduction of enhanced recovery care but it is unclear what proportion of hospitals have introduced it. We estimate that it will not have been introduced effectively in more than 50% of UK hospitals to date.

Given the significant shortening of hospital stay and decrease in complications resulting from the use of an Enhanced Recovery Programme (ERP), can laparoscopic surgery further improve recovery? Two small single centre studies from Denmark and England [10,19] provided conflicting results. The Danish study reported 3 day median postoperative hospital stays in both arms, whereas the English trial showed a significant reduction in median hospital stay and readmission rate for the laparoscopic group. It is, therefore, unclear whether optimal results following elective colorectal resection are achieved by laparoscopy or by using open surgery which has been optimized within an Enhanced Recovery Programme. To answer this question the EnROL trial has been designed to examine outcomes in a multicentre setting. This study examines the hypothesis that laparoscopic surgery within an ERP will provide superior postoperative outcomes when compared to conventional open resection of colorectal cancer within the same programme.

## Methods/design

### Study design

**Figure 1:**
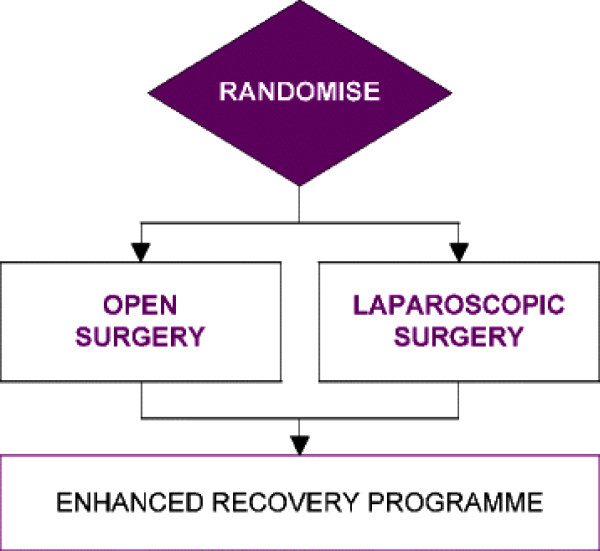
Randomisation schema.

The EnROL trial (Enhanced Recovery Open versus Laparoscopic) is a phase III, multicentre, randomised controlled trial, with blinding of patients and outcome observers during the first week post-operatively or until the date of postoperative discharge if earlier. Patients will be randomised between open and laparoscopic surgery, with both groups treated within an ERP, see Figure 1. The trial is being run at 12 centres in the UK which encompass a mix of rural and urban populations, treated within district general and specialist hospitals. In contrast to most previous laparoscopic colorectal trials the surgeons will have extensive experience of both laparoscopic and open surgery, having completed at least 100 laparoscopic colorectal resections and 50 open total mesorectal excisions prior to participating in the study. This should minimise the number of operations that will need to be converted to the open approach. Additionally surgeons will be provided with video recordings of standardized laparoscopic procedures prepared for the UK laparoscopic colorectal preceptorship programme.

The primary endpoint is patient reported physical fatigue measured by the physical fatigue domain of the multidimensional fatigue inventory 20 (MFI-20) [20] at 4 weeks after surgery. The secondary endpoints include postoperative hospital stay; 30 day and in-hospital complications; 30 day re-admission and re-operation rates; the other four domains of the MFI-20; a generic and cosmetic health related quality of life assessments [21-23]; observer measures of performance [10,24] and measures of resource use.

The study population is patients of age 18 and above who require elective resection of colonic or rectal cancer and are suitable for laparoscopic surgery. Exclusions to trial entry are acute intestinal obstruction, previous complex laparotomies, emergency admission and pregnancy. Patients are also ineligible when, in the opinion of the treating surgeon, conversion to open surgery is likely (such as in rectal cancer when preoperative imaging suggests tumour is present at the resection margin – ‘threatened margin’). Preoperative identification of the inability to insert an epidural catheter is also an exclusion criterion, as it would prevent patients who require it receiving postoperative epidural analgesia.

The EnROL trial has been approved by the National Research Ethics Service Committee South Central - Oxford B (REC reference: 07/H0605/150). The trial is co-ordinated by the Oncology Clinical Trials Office (OCTO) at the University of Oxford, with statistical support from the Centre for Statistics in Medicine, which together form Oxford Clinical Trials Research Unit (OCTRU). OCTRU is a UKCRN Registered Clinical Trials Unit. The trial is sponsored by the University of Oxford and North West London Hospitals NHS Trust. The Data Safety and Monitoring Committee and Trial Steering Committee, which includes a patient representative, will meet regularly in order to oversee the trial appropriately. Funding has come principally from the Bobby Moore Fund, Cancer Research UK (CR-UK) (Ref number: CRUK/07/019). Ethicon Endo-surgery have provided additional funding to facilitate provision of wound dressings and collection of pathology material.

### Randomisation

Following consent, eligible patients will be randomised in a 1:1 ratio to open or laparoscopic resection using a central computer system at the Oncology Clinical Trials Office. Simple randomisation will be used for the first 50 patients then minimization with a random element (0.8) [25]. The stratification factors used in the minimization are hospital, cancer site (colon/rectum) and age (<66 years, 66–75 years, >75 years). To facilitate blinding of outcome observers the randomisation allocation will be sent directly to the patient’s surgeon via email and will not be made available to the site staff member completing the randomisation process.

### Intervention

Surgery is to be carried out by one of the centre’s trial-accredited surgeons no later than 6 weeks after randomisation.

Surgery should be performed in a standard fashion by the trial surgeon(s) at each site, the only difference being the method of access. Rectal tumours within 10 cm of the anal verge should be treated by total mesorectal excision accompanied, whenever possible, by preservation of the hypogastric nerves. Tumours are defined as being rectal when at or within 15 cm of the anal verge on rigid sigmoidoscopy, performed with the patient awake on their left side. Conversion is defined as the inability to complete the dissection fully laparoscopically, including the vascular division, and it usually but not always, requires the use of a larger incision than that needed to remove the specimen.

### Blinding

Patients and outcome observers are to be blinded to the randomisation allocation until 7 days after surgery or the day of discharge if earlier. To facilitate this large Allevyn™ adhesive dressings will be provided for all trial patients. In addition, centres should ensure that all patient records which detail the randomisation allocation are stored in a sealed envelope within the patient’s notes until after the patient is unblinded.

Blinding is an important design feature of randomised controlled trials, reducing the risk of several forms of bias including reporting bias and observer bias. A successfully implemented blinding protocol improves the validity of trial results, however the success of blinding is infrequently tested or reported in randomised controlled trials. To investigate the role and success of blinding in surgical randomised controlled trials, and its impact on patient experience, the trial protocol was amended to include collection of quantitative data about the success of blinding using the Bang Blinding Index [26]. This statistical measure calculates the proportion of un-blinded patients (and research staff) in the trial by asking them to state to which arm of the study they believe they (their patient) have been allocated.

### Enhanced recovery care

The standardized perioperative care protocol (Enhanced Recovery Programme) is outlined in Table 1. It is possible for individual centres to amend this programme provided the treatment is standardized irrespective of randomisation, and the Trial Office is notified prior to the centre opening to recruitment. Compliance with the components of the Enhanced Recovery Programme will be recorded for all patients.

**Table 1:** Standardised Enhanced Recovery Programme for the EnROL Trial

Before admission		Conditioning of expectations of patient and carer by receipt of oral and written information at a dedicated preadmission visit, or by telephone counselling, with provision of a dedicated booklet or video sent by post.
		Meeting with stoma nurse if stoma anticipated.
		Identification of factors that might delay discharge and consideration of solutions e.g. provision of support when discharged if living alone.
		Co-morbid risk assessment: optimised pre-morbid health status.
Day before surgery		Avoidance of oral bowel preparation except in patients undergoing total mesorectal excision (TME) and reconstruction.
		Nutrition: three high protein/high calorie drinks if receiving oral bowel preparation.
Day of surgery	Pre-operatively	Preoperative oral carbohydrate loading to be given 2-4 hours prior to anaesthesia, using 200ml of fluid containing 12.5g/100ml CHO with a proven safety profile.
		Avoidance of long acting sedative medication from midnight prior to surgery.
	In theatre	Activation of thoracic epidural (T6-11) prior to skin incision.
		Avoidance of abdominal drains at primary operation.
		Avoidance of nasogastric drainage in the immediate postoperative period.
		Total volume of IV fluid < 3000ml.
		The use of upper body forced air heating intraoperatively.
		Local anaesthetic infiltration to the largest wound in minimal access surgery.
		Open surgery: small transverse or curved incisions when possible.
	After theatre	Oral intake of ≥ 800ml fluid (including oral nutritional supplements) postoperatively on the day of surgery, before midnight.
		≥ 200ml oral nutritional supplement postoperatively on the day of surgery, before midnight.
		Mobilisation by walking or sitting in a chair.
First Postoperative day from midnight – midnight (Day 1)		≥ 2 units of oral nutritional supplement taken.
		Termination of IV fluid infusion.
		Intake and tolerance of solid food.
		Intake of lactulose or a magnesium preparation to enhance bowel movements.
		Use of thoracic epidural analgesia.
		Mobilisation (out of bed) for at least 6 hours.
		Provided patient mobile, termination of urinary drainage on day 1, except after TME when it may be preferable to leave it until day 3
		Assisted mobilisation – 4 × 60m walks.
Second Postoperative day from midnight – midnight (Day 2)		Pain relief: termination of the thoracic epidural analgesia.
		Use of a multi-modal analgesic regime at, or before, discontinuation of thoracic epidural analgesia e.g. paracetamol and non steroidal anti-inflammatory or equivalent.
		Termination of urinary drainage on day 2 or earlier, except after TME when it may be preferable to leave it until day 3.
Discharge		Aim for discharge day 2-3 for colonic and proximal rectal resection; day 5 when a stoma fashioned.
		Discharge Criteria: patients must be tolerating normal food, mobilising independently and be managed on oral analgesics to fulfil discharge criteria.
		Follow up: provision of hospital contact numbers to allow discussion of problems; expedited review on ward if problems within 2 weeks of surgery.
		Review in out patient clinic at two weeks post operation.

### Measurement of outcomes

#### *Primary endpoint*

The primary endpoint is physical fatigue 4 weeks after surgery as measured using the physical fatigue domain of the multidimensional fatigue inventory 20 (MFI-20). The MFI-20 is a 20-item self-report instrument designed to measure fatigue. It covers the following dimensions: general fatigue, physical fatigue, reduced activity, reduced motivation and mental fatigue.

#### Secondary endpoints

### Postoperative hospital stay

Postoperative hospital stay will be reported, counting the day of operation as day zero.

### Complications, re-admission, re-operation and mortality rates

30 day morbidity will be assessed at 30 days post-operatively by the centre’s Research Nurse using a standardised definition of complications which have been modified from Lang et al. (2001) who reported a doubling of cost and hospital stay associated with postoperative complications [27]. The standard definitions of complications are detailed in Table 2. 30 day re-admission and re-operation rates, along with 30 day and in-hospital mortality will also be recorded.

**Table 2:** Standard definitions of complications for assessing morbidity of trial patients

**Category**	**Definition**
Cardiorespiratory	Respiratory failure - requiring mechanical ventilation
	Cardiac failure: cardiac index < 2 litres per m^2^ (treated first by fluid resuscitation and if no response by inotropic or vasoconstrictive medication)
	Pulmonary oedema - radiological diagnosis
	Arrhythmia - ECG changes requiring medical treatment and/or electroconversion
	Pleural fluid - radiographic diagnosis
	Acute myocardial infarction - electrocardiographic diagnosis
	Acute renal failure - requiring haemofiltration
	Stroke with neurological symptoms
	Pulmonary embolism
	Distal ischaemia
	Deep vein thrombosis - requiring duplex, radiological or other confirmation
	Other cardiorespiratory
Surgical	Unexpected blood loss >0.5 litres during operation
	Bowel perforation
	Ureteric damage
	Wound dehiscence involving separation of deep abdominal wall closure
	Postoperative bleeding - overt blood loss requiring > 2 litre transfusion with a normal clotting profile.
	Delayed oral intake - intravenous fluids > 1 week owing to postoperative ileus
	Bowel obstruction requiring reoperation
	Anastomotic leakage defined within 30 days of surgery radiologically (demonstration on abdominal CT with oral contrast, MRI or by contrast enema), surgically (visual evidence of faecal leakage at reoperation) or at autopsy (presence of a disrupted anastomosis).
	Necrosis of stoma - requiring surgery
	Aspiration Pneumonia - radiological diagnosis with appropriate history
	Other surgical
Infective	Sepsis - pyrexia > 38 ^o^C, septic focus or positive blood culture
	Postoperative peritonitis - clinical diagnosis
	Abdominal abscess - ultrasonograpy, computed tomography or operative diagnosis
	Necrotising fasciitis
	Wound infection - defined as any one of the following: (modified from reference 26)
	1. Purulent discharge or the aspiration of pus
	2. Erythema or localised swelling requiring antibiotics or surgical drainage, unless the drainage is clear and negative on culture i.e. a seroma
	3. A diagnosis of a wound infection made by a doctor.
	4. Report of wound discharge by the patient unless it is proven to be uninfected
	Chest infection - radiological diagnosis or empyema
	Urinary tract infection
	Disseminated intravascular coagulation
	Other infective complication

Major morbidity is defined as any of the following occuring within the hospital admission or 30 days of surgery: haemorrhage (requiring transfusion), any re-operation or readmission, anastomotic leakage, wound dehiscence, sepsis requiring at least high dependency support, HDU stay of > 5 days, unplanned admission to Intensive or Coronary Care Unit and death.

### Patient reported and functional outcomes

Health related quality of life will be assessed using the SF-36 questionnaire [21]. Quality of life will also be analysed with the EQ-5D. Cosmetic outcomes will be performed using a body image assessment [23,28]. Time points for the completion of these questionnaires are shown in Table 3.

**Table 3:** Time points for the collection of patient reported and functional outcome data

**Questionnaire/Assessment**	**Data Collection Time Point**				
	Pre Operative*	**Post-operative**			
		**4 weeks**	**3 months**	**6 months**	**12 months**
MFI-20	X	X	X	X	
SF-36	X	X	X	X	
EQ-5D	X	X	X	X	
Health Economics	X	X	X	X	
Body Image	X			X	X
SPIs	X	X	X	X	

^*^ To be completed not more than 4 weeks prior to surgery.

An observer assessment of physical function will be measured using standardized, objective performance indicators (SPIs) [24] which comprise a test of lower limb strength, balance and endurance. SPIs will be measured prior to surgery and then at 4 weeks, 3 and 6 months postoperatively. The tests differs from those described by Guralnik et al., in that patients will be measured for walking speed along a 10 metre, instead of 8 foot course, with a 61.5 cm (2 foot) clear zone at each end as originally described.

### Health economics outcomes

Health Economics questionnaires and EQ-5D will be completed by all patients providing information about the use of healthcare post-operatively for 6 months. Data collected will include GP surgery visits, GP home visits, in- patient stays, out-patients visits, District Nurse visits, physiotherapy and stoma nurse usage. Data will also be collected from the trial records to calculate the relative cost of the operative and peri- operative periods. Costs to be calculated include in-patient hotel costs, in-patient treatments costs (including re-admission and re-operation within 30 days and convalescent care), operating theatre costs and drug costs [29].

### Other domains of the MFI

The other four domains general fatigue, reduced activity, reduced motivation and mental fatigue will be analysed.

### Follow up & data collection

Patients will be seen at 2 and 4 weeks and then at 3 and 6 months post operatively. Follow up data will also be collected at 12 months after surgery. Research staff at the centres will collect the data and submit it to the Trial Office via the EnROL electronic data capture website.

### Pathology

Resection specimens will be photographed to judge the quality of surgery of the mesorectum [30], anal sphincters, [31,32] and the mesocolon [33][34]. Dissection will be carried out according to the trial protocol using cross sectional slicing, careful inspection for distance of extramural spread, lymph nodes, extramural vascular invasion and peritoneal involvement. TNM version 5 and modified Dukes grading including C2 and stage D will be used and regression grading and data collected by protocol. The distance to the high tie from the tumour will be recorded. Quality will be assessed locally and centrally with the addition of measurements of the specimens’ physical characteristics performed centrally. The quality of laparoscopic and open surgery will be compared to previous studies to facilitate assessment of the results of the trial.

### Statistical analysis

Sample size: The original sample size calculation required 266 patients (133 per treatment arm) to allow 90% power to detect a difference in the primary outcome of fatigue (as measured by physical fatigue domain of the MFI-20) of 0.45 standard deviations at p = 0.05 (two-sided). This includes a loss to follow up of 15% and a conversion rate of 8% from laparoscopic to open surgery. Following slower than expected recruitment the power was lowered to 80% in order to complete the study in a timely manner. With all other variables remaining the same the subsequent sample size of 202 (101 per treatment arm) is required. Additionally the study is powered to detect a 3 day difference in hospital stay at 80% power and p = 0.05 (two-sided).

### Analysis

All analyses will be on an intention-to-treat basis.

The primary outcome will be physical fatigue as measured by the physical fatigue domain of the MFI-20 at four weeks post operatively. If the primary outcome is normally distributed it will be compared using a *t*-test, ANCOVA adjusting for the minimization factors (primary analysis) and ANCOVA with further adjustment for prognostic factors.

Physical fatigue will also be compared at three and six months, the remaining quality of life variables at 4 weeks, 3 and 6 months, and the cosmetic outcome at 6 and 12 months, using the same analyses as for the primary outcome. Physical fatigue will be adjusted for the level of physical fatigue prior to surgery. The other measures of fatigue will also be reported.

For all continuous outcome variables, if there is severe departure from Normality the first approach will be transformation. If the data cannot be transformed to Normality, a Mann–Whitney test will be used. In the latter case no adjustment will be made for the minimization or prognostic factors.

Hospital stay will be compared using Kaplan Meier plots and log rank tests. The complications, re-operation and re-admission data will be binary and will hence be compared between the randomisation groups using a chi square test (or Fisher exact test if the data is sparse).

Quality of Life assessments at 4 weeks as measured by the SF-36 will be analysed as suggested in the SF-36 manual, comparison between treatments will use *t*-test and ANCOVA to adjust for prognostic factors. Mixed-effects models of repeated measures will be used to evaluate longitudinal comparisons (4 weeks, 3 months and 6 months).

The other four domains of the MFI will be analysed in the same way as the physical fatigue domain specified in the primary analysis.

The comparison between treatment groups of standardised performance indicators will use t-tests and ANCOVA adjusting for prognostic factors and baseline.

The tumour stage, quality of surgery grade, the physical measurements and the frequency of Dukes’ C2 cases will be compared between groups.

Analysis of the health economic data will be undertaken separately by those involved in the health economic analysis. Costs will be estimated from the perspective of the UK NHS and health benefits expressed in terms of quality-adjusted life-years (QALYs). The cases where laparoscopic surgery may be considered cost effective will be pre-defined and the mean differential costs and QALYs will be calculated in order to assess whether any of these conditions are satisfied. The relative costs of the operative and peri-operative period will be derived for all trial patients including the cost of consumables that are likely to differ between the two surgical procedures.

QALYs will be calculated for each patient in the trial, on the basis of their responses to the EQ-5D pre-operatively, at one month, three months and six months. Given that the time horizon of the analysis will be less than one year, total costs and QALYs remain undiscounted. To account for the skewed nature of the data, 95% CIs for the differential costs and QALYs will be estimated using bootstrapping methods. Missing data will be imputed using a multivariate multiple imputation procedure (Solas 3.0).

Statistical analysis will be undertaken using Stata (StataCorp LP).

## Discussion

The EnROL trial has been set up to examine the hypothesis that laparoscopic surgery improves outcome when compared to open surgery for colorectal cancer resection, even when both methods are optimized within an ERP. Prior to 2011 there had only been two small studies examining this issue [10,19] and they reported conflicting results. Other trials reporting an improvement in clinical outcome following laparoscopic surgery did not use an enhanced recovery programme to optimize the open group [1,2,4].

In 2011 the LAFA trial [35] published outcomes comparing the two approaches to surgery +/− an ERP. In this study of patients having segmental colectomy, total hospital stay was reduced following laparoscopic surgery when compared to open surgery. There are significant differences between the EnROL and LAFA trials that warrant continuation of EnROL. The EnROL trial only involves very experienced laparoscopic surgeons; it will include patients with rectal carcinoma; it will not include patients with benign diagnoses; and there will be no attempt to randomise between ER care and non ER care, as happened in the LAFA trial.

The involvement in this trial of surgeons experienced in laparoscopy is important as it minimises the % conversion rate to open surgery, thus providing a more homogeneous laparoscopic group of patients (given the intention to treat analysis) and increasing the potential to demonstrate differences in outcome. The choice of 100 prior laparoscopic resections to enable surgeons to contribute to EnROL is both intuitive and based on data from studies examining the acquisition of surgical competence with this approach [36]. Rectal carcinoma has been included in the EnROL trial as rectal resection is often more difficult than colonic surgery and also more demanding laparoscopically. As a result only one large randomised trial has been reported to date examining outcomes following resection of both high and low rectal cancers [6]. Generally hospital stay following rectal surgery is longer than after colonic surgery and therefore the potential to improve outcome after rectal surgery is greater, assuming laparoscopy decreases complications.

Patients with benign diagnoses have not been included in this study as in general they are younger and therefore fitter. By restricting the study to the treatment of cancer, and therefore an older age group who are generally less fit, it is likely surgery will have a greater impact on the patients - manifesting itself as a higher incidence of postoperative complications. The use of laparoscopic surgery has been shown to decrease postoperative complications [1,2] and the choice of only malignancy in the treatment group should highlight the potential benefits of this approach.

The primary endpoint of physical fatigue as measured by the Multidimensional Fatigue Inventory 20 (MFI-20) was chosen in order to provide a more global assessment of recovery than the normally used measure of postoperative hospital stay. In the past multicentre randomised trials looking at laparoscopic surgery have rarely found a difference in outcome when using a quality of life indicator. One exception was a trial of laparoscopic versus open donor nephrectomy that demonstrated a benefit to laparoscopy using both the MFI-20 and the SF-36 questionnaires [37]. The EnROL trial differs to many in that the surgeons are more experienced laparoscopic practitioners and thus we expect to see a lower conversion rate to open surgery and a more homogeneous ‘laparoscopic’ group of patients. The potential to detect a significant difference in outcomes will therefore be more likely than in some previous studies.

Currently laparoscopic colorectal resection costs more than open surgery because of the disposable equipment that is required. Many surgeons who practise conventional open colorectal surgery do not have the skills that would allow them to take on the minimal access technique and they will need extensive retraining in laparoscopy if they are to provide this new approach, which again has cost implications. In addition the procedure can take longer to perform than equivalent open operations, at least in the early stages of a surgeon’s laparoscopic experience. For all these reasons and also the potential for laparoscopic surgery to improve clinical outcomes when compared to open surgery, it is important that the EnROL trial examines this question within a multicentre setting.

## Abbreviations

EnROL, Enhanced Recovery Open versus Laparoscopic; MRI, Magnetic Resonance Imaging; LAFA, LAparoscopy and/or FAst track multimodal management versus standard care trial; ERP, Enhanced Recovery Programme; CR-UK, Cancer Research United Kingdom; CTAAC, Clinical Trials Awards and Advisory Committee; MFI-20, Multidimensional Fatigue Inventory 20; OCTO, Oncology Clinical Trials Office; SF-36, Short Form 36; EQ5D, Euroqol 5 Dimensions; SPIs, Standardised Performance Indicators; OCTRU, Oxford Clinical Trials Research Unit.

## Competing interests

Ethicon Endo-surgery have part funded the EnROL trial and RHK’s research. All other authors declare they have no competing interests.

## Authors’ contributions

RHK conceived of the study, participated in its design and organisation and drafted the manuscript. EAF is the Trial Coordinator and manages the day-to-day running of the trial and drafted the manuscript. SD provided statistical advice and drafted the manuscript. SL participated in the design and provided statistical advice. SRP is the Trial Management Director of OCTO and has overall responsibility for running of the trial at OCTO. JB participated in the quality of life aspects of the study and design. PQ designed the central pathology review aspect of the study. PJF designed the Health Economics aspect of the study. DJK participated in the design. All authors reviewed the manuscript, read and approved the final version. All authors read and approved the final manuscript.

## Pre-publication history

The pre-publication history for this paper can be accessed here:

http://www.biomedcentral.com/1471-2407/12/181/prepub
